# User, Usage and Usability: Redefining Human Centric Cyber Security

**DOI:** 10.3389/fdata.2021.583723

**Published:** 2021-03-10

**Authors:** Marthie Grobler, Raj Gaire, Surya Nepal

**Affiliations:** ^1^ CSIRO’s Data61, Distributed Systems Security, Melbourne, VIC, Australia; ^2^ CSIRO’s Data61, Distributed Systems Security, Canberra, ACT, Australia; ^3^ CSIRO’s Data61, Distributed Systems Security, Sydney, NSW, Australia

**Keywords:** cyber security, user, usability, usage, behavior, system design, human centric

## Abstract

The effectiveness of cyber security measures are often questioned in the wake of hard hitting security events. Despite much work being done in the field of cyber security, most of the focus seems to be concentrated on system usage. In this paper, we survey advancements made in the development and design of the human centric cyber security domain. We explore the increasing complexity of cyber security with a wider perspective, defining user, usage and usability (3U’s) as three essential components for cyber security consideration, and classify developmental efforts through existing research works based on the human centric security design, implementation and deployment of these components. Particularly, the focus is on studies that specifically illustrate the shift in paradigm from functional and usage centred cyber security, to user centred cyber security by considering the human aspects of users. The aim of this survey is to provide both users and system designers with insights into the workings and applications of human centric cyber security.

## 1 Introduction

The Internet belongs to its users with technology pervasive in most application domains, security considerations coming as an afterthought and usability of the security interventions seldom considered. Particularly with mobile devices being pushed into the centre of technology design, the current data centric Internet should adapt to focus especially on the user aspect (Conti and Passarella, 2018). To do justice to the benefits that can arise from the intended usage of technology applications, the focus should shift to keeping the users of the Internet safe from harm that may be caused by cyber security events. Although advances have been made in designing and implementing cyber security systems, human factors often still lead to their complete failure. Numerous examples exist in which users find ways to circumvent security measures put in place to protect them, or simply do not understand the dangers, despite well organised awareness campaigns (Kassicieh et al., 2015). Within the cyber security domain, there seems to be a constant trade-off between security requirements and accommodating human needs.

The understanding of cyber security and its boundaries used to be neatly defined within the boundaries of usage centred cyber security. However, the evolution of the Internet and associated technological advancements, combined with an ever changing way in which we use technology has again shifted our understanding of cyber security. In reality, the modern cyber security landscape is arguably without a perimeter considering the explosion in use of smart and mobile devices (Holland, 2020) and the rapid move toward a changed working environment as a result of the global COVID-19 pandemic (Whitty et al., 2020). Cyber security is no longer contained within organizational borders or home networks, but is present across borders, and reaching within the very basis of day to day life on an individual use level (Holland, 2020). This constant process of trade-off brings forth a security dilemma, in that many security mechanisms are put in place, but not adhered to, or in some cases actively circumvented by users (Kraus et al., 2017; Lebeck et al., 2018). It is from this security dilemma that the need for immersive security and a more in depth focus on human centric cyber security stems, necessitating a paradigm shift from functional and usage centred cyber security, to user centred cyber security.

Human centric cyber security is an intangible concept, difficult to define because of the inherent connection between humans and technology, and humans and security systems. For the purpose of this research, we consider human centric cyber security as involving all aspects of cyber security, with a particular focus on the human involvement in the system and processes. That is, understanding how humans represent value, but also risk to an organization; understanding how humans and computer interact and what risks are introduced as a result of these interactions. More importantly, understanding that the human can be regarded as both the point of success and failure, and that a specific trust relationship needs to be developed between the human and the system to ensure the correct balance (Holland, 2020).

The increased attention on a human centric design is the focus of contemporary research in cyber security. Particularly, the focus is shifting toward embedding human behavior and cognitive perception to ensure a fully human centric cyber security that not only protects humans and organizations from the harmful after effects of cyber security events, but do so in unison with human thinking and behavioral patterns. This stems from a clearer understanding that users (i.e., humans) alone are not solely responsible for the security of systems. Currently the norm is that designers of cyber security systems (i.e., the other humans) only focus on system aspects to defend against maliciousness. However, inherently, users of cyber security systems have diverse perception, knowledge and experience about the security risks that drive their behaviors. We argue that it is the failure of these humans, including architects, designers and developers, to acknowledge and duly consider the effect of different user attributes that is potentially the root cause of the problem. By conducting this review of human centric cyber security, we aim to identify the relationships of its different components and discover how those components work together as an interrelated and cohesive cyber security domain. This understanding will not only assist in lifting the general cyber security posture of humans, but also help to reduce the overall cyber security incidents.

In this exploratory survey, we consider the 3U’s of cyber security—user, usage and usability—to serve as foundation for understanding the relationships and inter-dependencies between the various cyber security components of a holistic cyber security view. We survey recent works in this field, and classify the developmental efforts based on their characteristics and identify future challenges in the development of human centric cyber security designs, implementation and deployment. Particularly, we focus on a number of studies that specifically illustrate the paradigm shift from functional and usage centred cyber security, to user centred cyber security by considering the human aspects of users. This survey aims to help system designers gain insights into these human aspects of cyber security and thereby implement successful cyber security programs, whilst supporting usability. Our findings include that the stigma around users as the weakest link of a cyber system are no longer the most prominent problem, but rather the disconnect between humans and the systems that they are depending on.

The rest of this paper is organised as follows. Section 2 provides context to the survey and an initial classification of the literature reviewed. Section 3 provides an overview of the move toward a more human centric (user) approach to cyber security, whilst Section 4 discusses the traditional system and procedural (usage) approach to cyber security. Section 5 discusses the human-system interaction (usability) aspects that are more conducive of cyber security success. Section 6 presents an illustrative case study in understanding the 3U model, with Section 7 concluding the study.

## 2 Human Centric Cyber Security Context

An important question to answer in the quest for true human centric cyber security is how to achieve national security and human security at the same time. To achieve these dual goals, we first classify existing security solutions into two groups. In *outward looking solutions*, the focus is on the threat actors, whereas in *inward looking solutions*, the focus is on the vulnerabilities of the cyber system (Cavelty, 2014). Then we can develop a holistic cyber security program that considers not only inward looking systems and procedures that are designed to protect users and organizations, but also outward looking humans with diverse cognitive biases, behavioral patterns and psychological needs and their interaction with the systems.

Human centric cyber security as a domain is still being developed and not well understood. It is only recently established as an amalgamation of traditional cyber security principles and the integration of human computer interaction, and with an increased focus on collaborative intelligence, with humans and technology working alongside each other. The domain shows promise as a socio-cognitive-technical approach to cyber security, focusing not purely on the role that humans play in cyber security, but developing a varied approaches that could ultimately lead to a balanced cyber security perspective, with not a single isolated point of failure. This is in contrast with the adage that “humans are the weakest link in a technical system.” In establishing a foundation for the concept of human centric cyber security, we have selected three components of cyber security for consideration—3U’s (user, usage and usability)—in designing, implementing and assessing cyber security systems. These components are not exhaustive representations of human centric cyber security, but regarded as of particular importance since it represents the multi-dimensionality of the cyber security context. In establishing this domain, we consider, as a starting point, the technical aspects and functionality of the system design, the human user engaging the system with the intended functionality in mind, and the associated thought and behavioral process by the human whilst interacting with the system for the intended functionality.

**FIGURE 1:**
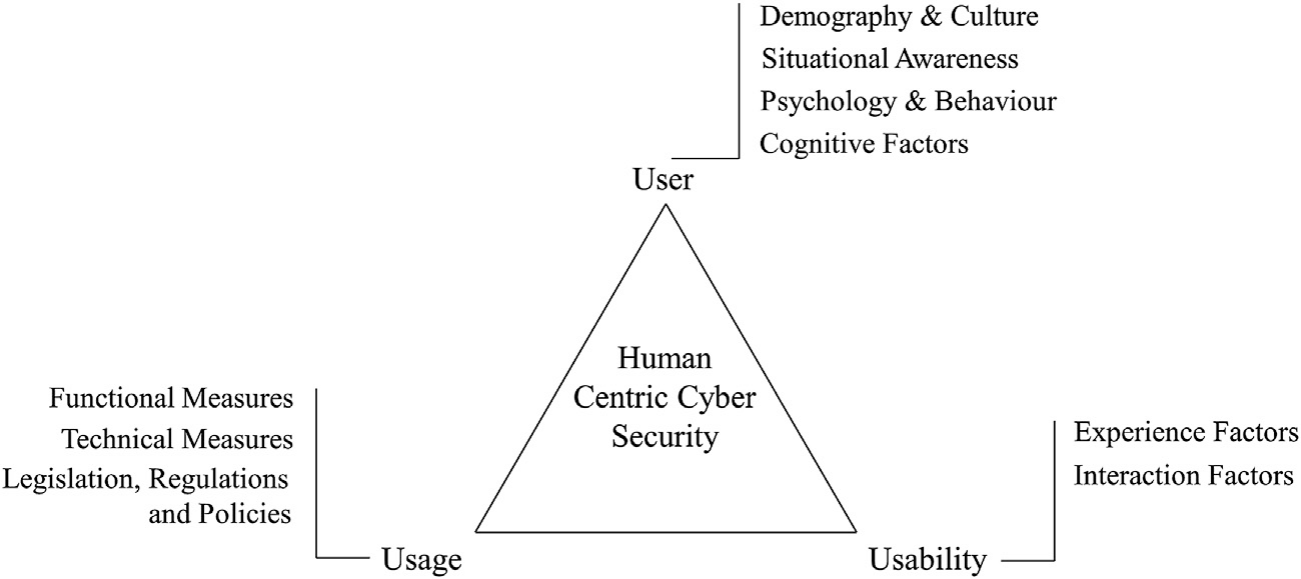
3U’s of human centric cyber security.

The 3U’s are specifically selected as foundation for this review. Figure 1 presents an overview of the different components of human centric cyber security and shows the overall scope of the paper. **User** components consider the human who interacts with the cyber systems for legitimate purposes. The diverse range of these users, all with different levels of cyber security awareness due to personal influences, demography and past experiences, as well as the user psychology and behavior toward cyber security risks are considered in the user component of cyber security. **Usage** components are mainly concerned with the functional aspects of technological and non-technological measures that are put in place to protect users against known security threats (Kraus et al., 2017). It focuses on the intended use of a cyber security system and mechanisms such as antivirus programs, spam detection algorithms, password based authorizations, organizational policies and cyber security laws. which has been a major focus of security researchers. Finally, the **usability** components consider how well the system can be used by the actual user. It presents an understanding of humans’ interaction with technology, and considers the interplay between the user and the product that they are using. It includes aspects of non-functional factors such as aesthetic and affective aspects of human computer interaction (Kraus et al., 2017), as well as a further familiarity beyond traditional user experience where the user is immersed in the relevance and application of security.

To understand the context of human centric cyber security as a socio-cognitive-technical approach, consider an example of personalised access of information requiring user authentication. Authentication is performed by systems requesting a username and password combination from users. Policies are developed to enforce the password length (e.g., minimum 8 characters) and complexity (e.g., requiring letter case, numbers, special characters, etc.). Furthermore, illegal access to the information is prohibited by law. However, passwords are created by users who may decide to use the same password for different websites, or share them with others. These behaviors depend on users’ personal attributes such as demography, situational awareness, psychology and cognitive factors. Therefore, the authentication should be performed in a usable way considering user attributes without creating a burden on the user. For example, multi-factor authentication techniques can reduce risks arising from user behavior by adding complexity to the process. Alternatively, biometrics based authentication can remove the requirement for passwords, although such approaches can have their own limitations.

To address these aspects, researchers have proposed different methods and design architectures. We conducted an exploratory literature survey into advancements made in the development and design of the human centric cyber security domain as summarised in Table 1. This survey was not intended to be an exclusive and exhaustive representation of the domain, but aimed at providing an overview and better understanding of current progress and advancements, as well as a clearer view of what the domain boundaries could be. To capture a reasonable range of literature on the emerging domain, we used ACM and IEEE databases, Google Scholar and DBLP as the primary sources.

**TABLE 1:** Summary of human centric cyber security literature surveyed.

User		
Demography and Culture (Section 3.1)	Users’ social and economic characteristics, combined with a shared set of attitudes, beliefs and practices influence the user’s personal experience. Relevant studies may help identify factors that contribute to increased online risk behaviors and thereby develop mitigation strategies	Camp et al. (2019); Carrol (2013); Da Veiga (2016); Higashino and Uchiyama (2012); Jeong et al. (2019); Leenen et al. (2018); Liu et al. (2014); Renaud and Flowerday (2017); Ruoti et al. (2015); Simko et al. (2018); Tam et al. (2010); Ur et al. (2015); Van Schaik et al. (2017)
Situational Awareness (Section 3.2)	Education and training may improve users’ awareness and perception pertaining to the cyber security situation with respect to time and space, comprehension of meaning and the projection of potential future implication. Gamification and immersive experience could be more effective for creating better situation awareness	Adams and Makramalla (2015); Bosnjak and Brumen (2016); Grobler et al. (2011); Howard and Cambria (2013); Jones and Endsley (1996); Kassicieh et al. (2017); Kluge (2007); Labuschagne and Eloff (2014); Newman et al. (2002); Renaud and Flowerday (2017); Tam et al. (2010); Tan and Aguilar (2012); Van Schaik et al. (2017)
Psychology and Behavior (Section 3.3)	Human psychological needs such as autonomy, competence, security, stimulation and keeping the meaning may affect users’ behavior toward cyber threats and use of cyber security systems. Systems that fulfill both psychological and security needs may perform better compared to ones that fulfill security needs alone	Abbott and Garcia (2015); Adams and Sasse (1999); Bonders and Slihte (2018); Bonneau and Preibusch (2010); Devillers (2010); Dourish et al. (2004); Gcaza et al. (2017); Kraus et al. (2017); Moller et al. (2012); Pfleeger and Caputo (2011); Stobert and Biddle (2014); Tam et al. (2010); Thomas and Galligher (2018); Tischer et al. (2016); Ur et al. (2015); Van Schaik et al. (2017)
Cognitive Factors (Section 3.4)	People have different memory recall and recognition capabilities. They make choices to balance security and complexity, their risk appetite in digital lives may differ from their physical lives, and their actions may not align with their stated beliefs. Understanding users’ cognitive factors may help select and/or develop systems that work for them	Camp et al. (2016); Devillers (2010); Dourish et al. (2004); Gao et al. (2018); Hadlington and Murphy (2018); Pfleeger and Caputo (2011); Pilar et al. (2012); Ruoti et al. (2017); Stobert and Biddle (2014); Tam et al. (2010); Ur et al. (2015); Williams et al. (2017)
**Usage**		
Functional Measures (Section 4.1)	Cyber security systems have defined functions to protect users and organizations, but they communicate with technical jargon. Considering general end-user in the context of social and physical environments may improve the effectiveness of the intended functionality	Felt et al. (2012); Giacobe and Xu (2011); Iacono et al. (2017); Kraus et al. (2017); Moon et al. (2015); Qu et al. (2014); Renaud and Flowerday (2017); Wu et al. (2018)
Technical Measures (Section 4.2)	Technologies are promised to provide transparent security, making users unaware of security threats. Developers of these technologies may not be aware of the flaws of their systems which could be exploited by adversaries	Acar et al. (2017); Bonneau and Preibusch (2010); Chiasson et al. (2006); De Donno et al. (2018); Florencio and Herley (2007); Gerber et al. (2017); Grobler et al. (2012); Gunson et al. (2011); Julisch (2013); Kraus et al. (2017); Komanduri et al. (2011); Stobert and Biddle (2014)
Legislation, regulations and policies (Section 4.3)	Multi-jurisdiction span of cyber space makes legislative instruments less effective against cyber crimes. Security policy frameworks are cognitively complex to implement effectively	Acar et al. (2017); Cavelty (2014); Grobler et al. (2012); Julisch (2013); Raschke et al. (2017a); Swart et al. (2014); Weber (2010)
**Usability**		
Experience Factors (Section 5.1)	Transparency of cyber security systems makes it difficult for legitimate users to comprehend the design of the system, leading to resistance and circumvention. Users implementing security systems experience difficulty in deploying them. Considering immersive user experience may help with compliance and effectiveness of cyber security measures	Aumi and Kratz (2014); Braz and Robert (2006); Greaves and Coetzee (2017); Hadlington and Murphy (2018); Iacono et al. (2017); Julisch (2013); Kraus et al. (2017); Krol et al. (2015); Lebeck et al. (2018); Pfleeger and Caputo (2011); Stobert and Biddle (2014); Tam et al. (2010)
Interaction Factors (Section 5.2)	Users’ interaction with cyber security should be intuitive, understandable and comfortable. Understanding their interactions with the entire technology ecosystem could make cyber security systems more usable	Agarwal et al. (2016); Bonneau and Preibusch (2010); Conti and Passarella (2018); Denning et al. (2009); Dunphy et al. (2014); Gerber et al. (2017); Haack et al. (2009); Hassenzahl et al. (2010); Higashino and Uchiyama (2012); Kraus et al. (2017); Lebeck et al. (2018); Rajkumar et al. (2010); Riahi et al. (2014); Senarath et al. (2019); Tyworth et al. (2013); Wijayarathna et al. (2017); Wu et al. (2018)

We focused particularly on articles identified with keywords including *human centric cyber security, usability, user behavior*, and *usable security*. We further applied a chain sampling approach, identifying and searching for non probabilistic articles of relevance in the initially identified articles’ reference lists. We specifically focused on articles published between 2005 and 2019. In addition, only peer reviewed articles published in English were considered. All articles of which the full text was not fully accessible were automatically excluded from the review. Editorials, position papers, keynotes, and panel discussions were also excluded. Of the 111 articles obtained, only those articles that specifically focused on a human centric cyber security element were considered, resulting in the final inclusion of 78 articles. The data survey, extraction and classification of the literature were performed by three reviewers between August 2018 and December 2019.

We will review 3U components of human centric cyber security in more detail in following sections, starting with the user component in the next section.

## 3 User

Human vulnerabilities account for 80% of total vulnerabilities exploited by attackers, yet the focus of cyber security is often targeted only on system tools and technology (Adams and Makramalla, 2015). This section addresses behavioral measures of the individual users, awareness and training as indicators of cyber security culture, in addition to some involvement in terms of technical measures discussed in Section 4.2. The premise is that users believe themselves to be invulnerable to security risks, i.e., *“It will happen to somebody else”*, and therefore opt for convenience above security. By leveraging behavioral science, the immersive cyber security experience can be improved, providing valuable insight in terms of cognitive load and bias (Pfleeger and Caputo, 2011).

**FIGURE 2:**
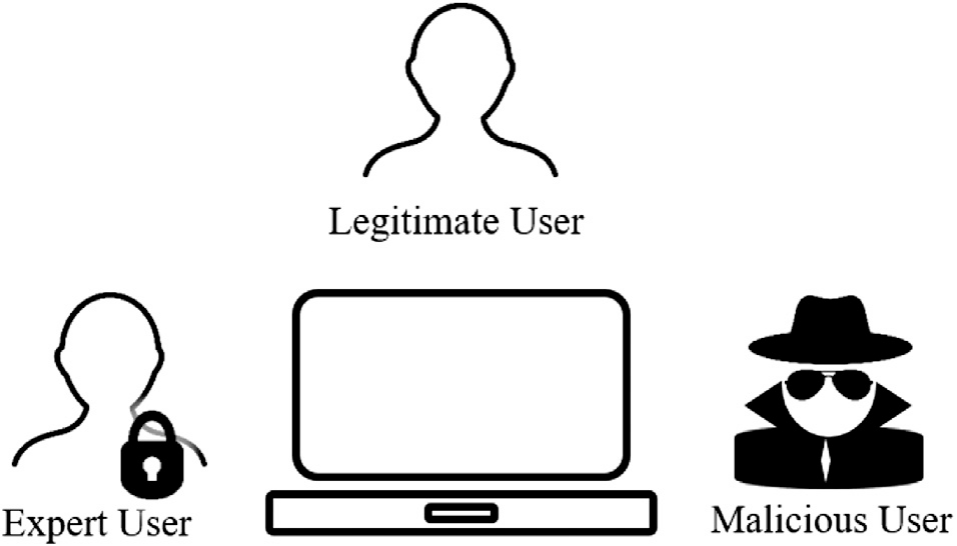
Cyber security: User perspective.

Figure 2 presents the user perspective of cyber security, where both the attack and the defence mechanism may be a hindrance from the user’s viewpoint. Although our research particularly focuses on the legitimate system user, we acknowledge the existence of different categories of users, including malicious users and specialised expert users. These identified users are not exhaustive, but provides an overview perspective of the user component. Here, we review four user components of cyber security: demography and culture, situational awareness, psychology and behavior, and cognitive factors.

### 3.1 Demography and Culture

Demographics carry relative weight in terms of socio-technical approaches and is a key component to address the interaction of human and technical aspects within a cyber environment. The user is assumed to be unpredictable in terms of applying security, as demonstrated through the different roles played by humans. Therefore, user behavior is studied to better understand and eventually improve security behavior. Although demographics do not play a leading role in the cyber security focus, its importance leads to its inclusion as a distinct dimension in the proposed health cyber resilience model (Camp et al., 2019). This model enables statistical characterizations to create an empirical yet realistic view of human behavior in establishing a global baseline for identifying and extrapolating factors that contribute to increased vulnerability to online risk behaviors.

Through our literature survey, we identified that both internal and external factors can have an impact on cyber security related behavior. Specifically, the impact of specific demographical factors has been studied on the ability of users to successfully use technical authentication methods. It is believed that a user’s personal experience can have a direct influence on their decisions made in cyber space (Tam et al., 2010; Camp et al., 2019), whether it relates to the passwords that are chosen or mannerisms and behavior stemming from other areas in their life. Limited studies focus on identifying user vulnerabilities specific to country or geographical domain (Liu et al., 2014; Ruoti et al., 2015; Ur et al., 2015; Van Schaik et al., 2017).

Culture can be defined in terms of a collective and shared sense of relatedness to human experience. In this context, insight into cultural differences, particularly from an organizational perspective, has become a critical priority within an increasingly interconnected world (Leenen et al., 2018). According to Da Veiga (2016), it is *“the intentional and unintentional manner in which cyber space is utilised from an international, national, organizational or individual perspective in the context of the attitudes, assumptions, beliefs, values and knowledge of the cyber user.”* It stems from the cyber security procedural knowledge and consistent application thereof by a cyber user.

Research has also been done in terms of cyber culture on a national level, and how that influences individuals’ behavior in cyber space. Particularly, a definite link was established between national culture dimensions and cyber security maturity, as a result of analysis of password practices. Analysis showed that the extent to which less powerful members in a society accept that power is distributed unequally (i.e., power distance index), the level of connection that society makes between the past, present, and future (i.e., long-term orientation) and the degree to which people prefer being left alone to look after themselves or want to remain in a closely knitted network (i.e., individualism), all contribute to the cyber security maturity level within a specific social group (Jeong et al., 2019). Although the link has been established on a national level, it is accepted that social identity is dynamic and that these cultural influences will remain fluid as the user moves between different social identity groups. These findings concur with research by Simko et al. (2018) that investigated the relations between culture and needs related to security and privacy. Although this study specifically focused on recently settled refugees in the United States through interviews and focus group studies, some key findings are very relevant in that common best practices are not always feasible and cannot be assumed as common knowledge. The study found that “traditional” security mechanisms like password based authentication are not designed for users from different cultural backgrounds, and that some security concepts like identity theft are not known to all.

### 3.2 Situational Awareness

According to Jones and Endsley (1996), situational awareness is a combination of information acquisition and interpretation to incorporate four stages: perception (acquiring available facts), comprehension (understanding facts in relation to our knowledge of such situations), projection (envisioning how the situation is likely to develop), and prediction (evaluating how outside forces may act upon the situation to affect the projections). Where humans are concerned, Howard and Cambria (2013) are of the opinion that situational awareness should be extended to also include intention awareness as the process of integrating actors’ intentions (both the user and the system) into a unified view of the surrounding environment. This concurs with the generally accepted user model, where the legitimate users aim to respectively defend or use the system, whilst the malicious attacker’s intention is to attack. Many works have shown the influence of a person’s environment and exposure to the Internet in their online security behavior (Tan and Aguilar, 2012; Bosnjak and Brumen, 2016; Van Schaik et al., 2017). However, a further need was identified to develop cyber systems that incorporate or enhance existing situational awareness to not only address raw quantitative measures, but also include the full consideration of human actors and their unpredictable behaviors (Howard and Cambria, 2013).

In the cyber context, the appropriate reaction to cyber events require a high degree of situational and intention awareness, enabling the user to quickly adapt and even pre-empt unpredictable actions that may occur. The user should consider a large scope of environmental and informational attributes (Howard and Cambria, 2013). For example, a user should be able to look at the subject line and sender name of a received email (i.e., perception) and be sufficiently aware of their environment to make an informed decision on whether or not trust the email. *“Do I know the sender? Do I do business with the sender or with an institutional name? Is the subject line relevant to something that is currently pertinent in my life?”* (i.e., comprehension). The user should also be able to take this comprehension one step further to assess the intention of the sender to ascertain any potential cyber security implications that may arise (i.e., projection) if they proceed with clicking on the email to open it (i.e., prediction).

To further a user’s situational and intent awareness, security awareness frameworks exist which prescribe steps required to design and implement an effective security awareness program, refer to the European Union Agency for Network and Information Security (ENISA) and the National Institute of Standards & Technology (NIST) Awareness Framework. These programs aim to equip cyber users with the necessary knowledge to identify and mitigate threats (Labuschagne and Eloff, 2014). Although the importance of cyber security awareness training is clear (Newman et al., 2002; Grobler et al., 2011; Renaud and Flowerday, 2017), it does not always provide the necessary skills training required to better protect against cyber attacks (Adams and Makramalla, 2015). For instance, in a global survey of IT professionals, Dimensional Research (2018) found that 43% of them had seen their organizations targeted by social engineering schemes, despite social engineering often being a focus area in cyber security awareness contexts. Linked to the implementation of these frameworks, a variety of approaches can be used to further cyber security awareness and training campaigns.

Adams and Makramalla (2015) also found that cyber security training for all employees is inefficient in conveying the necessary knowledge and skills for employees to reduce the number of successful attacks. Therefore, the recommended approach to awareness and training is to keep users informed about current security issues (Tam et al., 2010), without overloading them with unnecessary technical information that does not contribute to understanding of the security problem. It also argued that awareness messages should utilise timing, location, and social coercion to keep the best practices fresh in mind, and innovative games to stimulate and provide feedback to improve interest in and memorability of cyber security concepts (Kassicieh et al., 2017). A more formal approach to cyber security training includes the focused support of training certification. Kluge (2007) suggests that organizations must take the lead in developing appropriate high-level principles for professional certification and security protocols and in harmonising these on a global basis. This will support the provision of a firm and consistent foundation for international treaties within the cyber security domain. Gamification is another modern approach of creating situation awareness, promoting active learning and motivation whilst increasing retention of the learned skills. This is in contrast with more traditional approaches such as instructor-led classes or information posters (Adams and Makramalla, 2015). Research by Kassicieh et al. (2017) found that cyber security training and awareness programs are limited in their efficacy if they are not targeted. from pyramidal mentorship training for a diverse workforce to velcro learning and flipped classroom techniques.

### 3.3 Psychology and Behavior

Cyber security is not just about technology, with findings by Dourish et al. (2004) indicating that users have neutral to negative attitudes toward security solutions. The reasons include security solutions being perceived as barriers to work, security extending from online environment to physical worlds, delegation of security to others including technologies, individuals and organizations, etc. For example, users rarely choose passwords that are both hard to guess and easy to remember. Accordingly, the origin of cyber attacks is often the vulnerability of the victim, rather the ingenuity of the attacker.

To determine how to help users choose good passwords, Tam et al. (2010) performed a controlled trial of the effects of giving users different kinds of advice. They found that the motives behind password selection and password management behaviors are complex, often differing depending on the type of account in question. They also found that the timeframe of password selection affects the motivation to choose a secure password. This timeframe refers both to the time in a person’s life during which the password needs to be formulated, as well as to the timeframe/regularity with which the password will be used, for example, daily, weekly, monthly, etc. A study conducted at Carnegie Mellon University after a change in password policy showed that users were largely frustrated with the change, but believed that the change made them more secure. These users adapted to find new coping strategies to handle their passwords management. Despite this, people are found to retain fragments of previous habits, such as using a root word as basis for all passwords, that results in long-term and extended password reuse (Stobert and Biddle, 2014). This cyber security misbehavior has been found to have a negative impact on cyber security culture.

The incorporation of an understanding of human behavior into cyber security products and processes can lead to more effective technology (Pfleeger and Caputo, 2011). In contrast, resistance to cyber security measures can compromise the effectiveness of the security level (Gcaza et al., 2017) and as such the psychological aspects should be considered in a true human centric cyber security design. Kraus et al. (2017) show that there is a relationship between personality traits and information security. They investigated the psychological need fulfillment of humans as motivators for security and privacy actions, specifically with smartphone usage. They identified 11 psychological needs that are met when using smartphones, with only one of these needed for security. They also noted that many of the actions may benefit security and privacy of the user, but the overarching need that is fulfilled is not security specific, therefore suggesting that security and privacy actions are considered more in terms of psychological needs fulfillment. From this study, they identify that the need for autonomy, competence, security, stimulation and keeping the meaningful are all salient as motivators for security and privacy actions. These psychological needs are discussed next in the cyber security context.

#### 3.3.1 Autonomy

The need for autonomy stems from feeling that a user is the cause of its own actions rather than the feeling that external forces or pressures are causing the actions. For example, Tam et al. (2010) found that users know what constitutes a good/bad password and know which common password-management practices are (in)appropriate. Still, they are motivated to engage in bad password-management behaviors because they do not see any immediate negative consequences to themselves and the general acceptance of the convenience/security trade off. Similarly, Moller et al. (2012) found that many Android app users did not immediately install updates, a behavior that may result in security vulnerabilities. Accordingly, the behavior of users should be adapted in consideration of the users’ cognitive view of the dilemma in order to frame this need in the cyber security context.

#### 3.3.2 Competence

This need stems from feeling capable and effective in a user’s actions rather than feeling incompetent or ineffective. Users often do not know the risks involved in specific cyber related actions, but do not want to appear incompetent. Tam et al. (2010) found that the time frame factor within password creation affects only more important accounts, such as an online banking account, where convenience/security trade off leans toward security. Therefore, users choose strong passwords only if they are willing to sacrifice convenience—understanding the importance of choosing a strong password alone is not sufficient. In a study, Tischer et al. (2016) suggested that users could be less willing to take risks and/or more willing to report security behaviors after they were explicitly told that they had fallen victim to an attack. This supports the notions that users do not want to feel incompetent or ineffective.

#### 3.3.3 Security

The need for security stems from feeling safe and in control rather than feeling uncertain and threatened by your circumstances. Users present overconfidence with an *“I won’t be affected by this”* attitude. Users have certain perceptions that can either positively or negatively impact their security process (Gcaza et al., 2017). Moreover, Van Schaik et al. (2017) show that poor security behaviors are adopted by users and maintained throughout their life progression (from students to workforce). Furthermore, a number of research studies state that poor security behavior is caused by the implementation of specific security mechanisms (Section 4), combined with users’ lack of knowledge (Adams and Sasse, 1999; Devillers, 2010; Abbott and Garcia, 2015). For example, despite research indicating the lack of user cooperation in terms of usable authentication (Ur et al., 2015), users can correctly evaluate password quality and identify poor password formulation strategies (Tam et al., 2010).

#### 3.3.4 Stimulation

The need for stimulation stems from feeling that a user gets plenty of enjoyment and pleasure rather than feeling bored and under stimulated. It is found that users often underestimate the risks associated with their behavior and therefore engage in risky behavior in order to be stimulated. For example, Tischer et al. (2016) conducted a study by dropping 297 flash drives across large university campuses and checking whether the attacks using such randomly dropped USB drives would be successful. The success rate was between 45 and 98% and the fastest successful attack was possible within 6 min 68% connected the USB drives to find the owner and 18% out of curiosity. The post attack surveys revealed that many of those users were motivated by altruistic behavior. They were not technically incompetent, but were rather typical community members who appeared to take more recreational risks than their peers, arguably out of the need for stimulation and pleasure.

#### 3.3.5 Keeping the Meaningful

This need stems from the urge to collect meaningful things. Several studies have identified interesting insights on techniques that users apply to create and keep track of accounts and passwords. Regardless of the additional measures put in place to ensure adequate security, human behavior leads users toward reusing the same or slightly changed password for multiple accounts, or multiple users using the same password (Stobert and Biddle, 2014). In this context, meaningful can simply refer to an account that would require more effort to reset in the event of forgetting a password. For example, many websites offer a reset password service that is fairly easy to reset vs. an online bank account password reset that may require the user to physically visit a bank branch with original identification documents in order to have the password reset (Gao et al., 2018). Another factor is the security recommendation to make backups of data (Bonders and Slihte, 2018; Thomas and Galligher, 2018), yet many victims of cyber crime often have no recent data backup to facilitate their recovery.

### 3.4 Cognitive Factors

Understanding how users typically react when faced with complex security situations is essential in designing usable security to prevent any cognitive biases from negatively impacting the cyber security application. To illustrate this, three specific examples of cognitive bias factors are presented.

**Example 1** Tam et al. (2010) show that 85.7% of users can correctly evaluate password quality, with 95.4% of users correctly identifying poor password formulation strategies. However, this theoretical knowledge does not translate into application, with a large proportion of users not choosing secure passwords to protect their accounts and devices, and an estimated 15% of all passwords containing a word or a name suffixed with the number “1” (Devillers, 2010). A study conducted by Ur et al. (2015) identified that users consider the use of a birth date or name as appropriate if they believe the information to not be readily accessible on social media. Many users also believe their own uniquely selected combination of dictionary words, or words with personal significance (such as a partner’s middle name) will prove random enough to evade guessing attacks.
**Example 2** In opinion polls, the public frequently claim to value their privacy but act contrary to this notion (referred to as the *privacy paradox* or *privacy calculus*). To illustrate this, Williams et al. (2017) hypothesise that Internet of Things (IoT) constrains protective behavior. They prove that the privacy paradox is significantly more prevalent in IoT, frequently justified by a lack of awareness (a third of respondents displayed an opinion-action disparity, i.e., saying that they do not trust IoT devices, but still buying the devices). The study found that IoT devices are considered significantly less private than non-IoT products. yet many users who recognised the risks still purchased the products.
**Example 3** To better understand users’ perceptions of their digital lives and how they manage their online security posture, Ruoti et al. (2017) conducted a series of semi-structured interviews with mostly middle-aged parents. They found that participants chose their security posture based on the immense value the Internet provides and their belief that no combination of technology could make them perfectly safe. The results revealed that participants’ misconceptions related to browser-based encryption indicators lead to insecure behavior—participants felt that secure email was less secure than texting because of its permanence. The paper refers to protection motivation theory, in which users react to fears by assessing the severity and probability of the threat and then appraising the efficacy of a recommended behavior and their ability to carry out that recommendation effectively, as an explanation of home computer users’ security behavior and motivating safe behavior online.

Hadlington and Murphy (2018) studied the relationship between media multi-tasking and risky cyber security behaviors of users. Their paper established a benchmark scoring mechanism for such behaviors, using the cognitive failures questionnaire to assess the lapses in cognition within the areas of perception, memory, motor function and the media multi-tasking inventory to capture the media use behavior. This study showed that individuals who engaged in more frequent media multi-tasking reported more everyday cognitive failures and a higher frequency of engaging in risky cyber security behaviors. Another important aspect of human cognition relates to memory ability, and the functionality of recall and recognition within the application of passwords. According to Camp et al. (2016) authentication using passwords requires three cognitively difficult actions. Firstly, a good password requires *generation* of a high level of entropy. Secondly, the person must reliably *recall* that highly entropic password. Thirdly, the person must properly *map* the password to the context. In their work, they envisioned the creation of a more usable system that can aid users in more accurately recalling their chosen passwords. Further studies by Stobert and Biddle (2014) and Gao et al. (2018) investigate the ability of users to remember their passwords, building on the ecological theory of memory and forgetting.

Gao et al. (2018)’s study sheds new light on password management, account usage, password security and memorability. They investigate the matter of memorability: *why do users remember some passwords, but not others?* The premise is that the depth of processing power (i.e., quickly generated passwords vs. passwords that were well thought out) and the encoding-retrieval match (visual cues when creating the password) have significant impact on memorability. Pilar et al. (2012) presented the results from a study performed to understand the limitation of human memory in terms of using passwords as authentication mechanisms. The interesting result is that the memory performance for passwords have direct links to the number of passwords rather than the age of the password. Gao et al. (2018)’s study supports this finding, presenting evidence of decay theory. They propose that human memory naturally adapts according to an estimate of how often a password will be needed, such that often used, important passwords are less likely to be forgotten. They also present the interference theory, which suggests that forgetting can be due to interference between similar memory traces such as when the passwords have similar words or are used in similar-looking applications. The study found the use of password managers for text-based passwords only at 1%, thus not solving the problem of forgotten passwords.

A study by Dourish et al. (2004) aims to understand the user’s experience on security as they use ubiquitous and mobile technologies in their daily life. The study examines users’ concerns about security, attitudes toward security, and the social and organizational contexts within which security concerns arise. The findings point particularly toward emerging technical solutions. The premise of the study is that effective security solutions depend not only on the mathematical and technical properties of those solutions, but also on users’ ability to understand them and use them as part of their work, as shown in this discussion on cognitive factors. This discussion on the psychological and behavioral aspects of users clearly show how the interactions between the user who use the system and the system designed to defend the user against attackers need to be integrated at all levels in order to present a fully human centric cyber security approach. The next section investigates the usage component in more detail.

## 4 Usage

**FIGURE 3:**
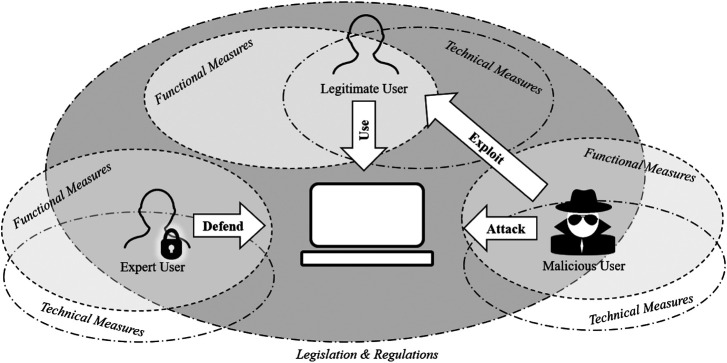
Cyber security: Usage perspective.

Usage focuses on traditional methods that encompasses the methods and techniques employed to increase the overall security of the system. As illustrated in Figure 3, the system’s functional design are based around defending the system against attacks. Both the system defence and the attacks may be driven by human actions. In this section, we review the three usage components of cyber security: functional measures, technical measures, as well as legislation, regulations and policies. These aspects work together, both as individual aspects and as a combined whole, to serve the intended usage of the system, but focused around security. For the purpose of this survey, we focus on the usage perspective of the legitimate user only.

### 4.1 Functional Measures

The main purpose of a security system can be regarded as the serving of a specific function, as stipulated or expected in a specific situation (demonstrated in Figure 3). From a simplistic user perspective, the function of anything within cyber space is to disseminate information required by the user and to alert the user when a cyber threat is detected. A legitimate user would expect the system to function as intended, and provide the necessary performance measures to enable the system to be used in a value added manner. Although we focus on the legitimate user of a cyber system, the same holds true for the malicious user who would be accessing information through the cyber system, exploiting legitimate users, and be on the lookout for intrusion detection systems that could alert against their own malicious attempts. Similarly, an expert user would make use of a range of informational sources to alert legitimate users in the event that a cyber threat is detected. In addition to these measures, a functional measure for a business is to maintain its continuity under attacks.

Information dissemination is one of the major functional aspects of cyber security in these technologies and can be regarded as the distribution of relevant information, as required, needed or specified. Renaud and Flowerday (2017) signaled the importance of using systems and technology to communicate both advanced and specialised informational concepts (such as the outputs of complex machine learning formulations), as well as more generalistic everyday type of information with users (such as a real time updated train table). A myriad of applications and technology resources exist that focus particularly on the continuous dissemination of information to users. These technological advancements provide users with convenience and offer corporations high-level business efficiency (Moon et al., 2015). However, not all information is presented in layman terms.

System generated security centric information are often hard to understand by general end-users. In general, they are too technical to be understood by ordinary users. Furthermore, users have varied linguistic preferences, which do not match the text. Wu et al. (2018) proposed an approach called PERSCRIPTION which aims to make system generated security centric descriptions in Android more understandable to the users. Their proposed approach helps users avoid malware and privacy-breaching apps by generating security descriptions that explain the privacy and security related aspects of an Android app in clear and understandable terms. Also in terms of information dissemination, visual representation is a valuable approach that considers both the usage and user components. To this end, Giacobe and Xu (2011) proposed a visualization tool for network security using GeoViz. The idea is to divide the whole IT network into zones and visualise the log data based on zones. Zones can be defined in an abstract level such as one zone could include desktops, and the other only servers, providing an automated visual view of technical information that also serves a functional purpose.

Although the intention is clear, it is possible that information sources are not maintained in terms of their currency and may become out of date. In addition, information may be presented in a complex manner, leaving the user unsatisfied and without a true understanding of the intention of the information that were disseminated. To illustrate the function of information dissemination, a study by Iacono et al. (2017) investigated the approaches to communicate the degree of over-privilege in mobile applications. It used an additional rating system in application stores to inform users before making the decision of installing a specific application. This system was evaluated in a usability study based on distinct prototype Android application stores. The findings show that passive security indicators can be applied to influence the decision-making process of users. Similarly, Qu et al. (2014) developed a system called AutoCog to automatically assess the descriptions against the need for permissions.

The design architecture of human centric computing environments enables users to access and use desired information from any place at any time by using human centric computing. This is possible because the computing environments are connected to networks everywhere. However, the important information available in these environments may equally always be vulnerable to malicious intrusion (Moon et al., 2015). To cope with cyber attacks, companies use network security equipment such as firewalls, intrusion detection systems, intrusion prevention systems, and security technologies such as antivirus software and data-loss protection. These technologies offer the capability for effective detection and response to known attacks because they use blacklists or signatures of known attacks. However, the usefulness of such protection methods is often questioned (Moon et al., 2015; Kraus et al., 2017). In mobile devices, the Android platform provides a permission system to inform users about the potential risks of installing an application. Felt et al. (2012) examined the effectiveness of the Android permission system at warning users and found that users pay little attention to these warnings, causing users to make incorrect security decisions. In the context of threat detection, current practices are often not effective despite the necessity of providing information about security matters. Presently, many security related decisions are required to be taken by general end-users, or even IT users with little security knowledge. For example, if home equipment such as IoT devices or home routers are set up, people are not expected to hire a security expert to do the necessary technical work. The general end-user needs to make a decision on several technical aspects such as encryption, frequency band, options, etc., whilst the systems are rather targeted for security expert users. Without appropriate user interfaces, these systems cannot properly interact with general end-users. Both these examples indicate that more work needs to be done to make the information dissemination usable.

### 4.2 Technical Measures

System security are often transparent to users, as shown in Figure 3. Technical approaches tend to free users from understanding the security details, but at the same time remove the transparency of the solutions so that users are unable to deal with the unexpected security situations. We specifically focus on cryptography and automation, since everyday activities have an increasing digital component requiring data secrecy and integration. It is becoming increasingly urgent to augment and automate cyber security in order to maximise outputs and minimise human error (Grobler et al., 2012). Within the context of usable authentication as one of the central themes in human centric cyber security, password based authentication are widely used in Internet systems. Bonneau and Preibusch (2010) studied the different practices employed in 150 different types of websites and found that poor practices were commonly deployed. For example, a lack of encryption to protect transmitted passwords, storage of clear text passwords in server databases and little protection of passwords from brute force attacks were common amongst the websites surveyed. In addition, several systems provide default login credentials which enables large scale attacks that can exploit default login credentials that many users never change, or long-term passwords that rarely change (De Donno et al., 2018).

Cryptography is the foundation of security, and most common cryptographic techniques are made available through software libraries. Improper use of cryptographic libraries are often the source of vulnerabilities. Acar et al. (2017) performed empirical studies of five cryptographic python libraries for its usability. They conducted a controlled experiment with 256 Python developers where they were asked to attempt common tasks involving symmetric and asymmetric cryptography using one of five different APIs. They observed that 20% of functionally correct tasks were not secure, although the developers believe that their code was secure. Therefore, although the usage component may be in place, the security aspect may not be addressed.

The automation of security has taken on momentum in recent times due to the popularity and success of Artificial Intelligence (AI)/Machine Learning (ML) techniques in achieving automation in different applications. This is critical for many organizations as they like to have a higher return on investment. On the one hand, there are not enough security experts to analyse security data, and on the other hand the data collected by security tools are growing. There are two key solutions to this: the first is to automate the tasks as much as possible using AI/ML tools, and the second is to increase the usability of security tools. This will enable targeted human centric cyber security that are more balanced in terms of the 3Us.

### 4.3 Legislation, Regulations and Policies

Legislation and regulation are important instruments to help deter cyber security offenses (see Figure 3). However, these instruments often do not seem to take the human into consideration. By mainly focusing on technicalities and national security, the individual user’s security concerns are often forgotten, causing a detrimental effect on the security of the whole system. The result is a security dilemma, i.e., the efforts by one actor to enhance security of a system effectively decreases the usability efforts by others. We specifically consider the legislation, regulations and organisation policies as applicable to the legitimate user. Due to the complexity of jurisdiction specific regulations and the differences in criminal law in terms of technology related offences, we acknowledge their existence but do not specifically investigate these specialisations in this survey.

Cavelty (2014) argues that the solution lies in security policies detailing anti-vulnerability and based on strong data security and privacy. These policies are driven by ethics of the infosphere that is based on the dignity of information related to human beings, also referred to as digital human rights. In addition, laws can only be enforced if authorities are made aware of infringements, hence the need for accurate threat detection and reporting. Compounding the problem is that an average data breach generally takes more than a month to be discovered. When companies take considerable time to discover (or do not know of) the breach, it creates a window of opportunity where the leaked data set is available for anyone to discover (Swart et al., 2014).

Another consideration within usage is whether a specific action is enforced by a rule specified within an authoritative normative document, such as an organizational policy framework, law or regulation. Lack of strong and enforced policies and human fallibility can cause unintended implications for users’ online privacy. An adequate legal framework must take the underlying technology into account and would best be established by an international legislator, which is supplemented by the private sector according to specific needs and thereby becomes easily adjustable (Weber, 2010). In the wake of the technological explosion, Grobler et al. (2012) recommended five elements that should be present in developing a national strategy for an effective cyber security approach and culture within the ambit of enforceable legislation: political will, adapted organizational structures, accurate proactive and reactive measures, reduced criminal opportunities, and education and awareness. Furthermore, the content of the developed legislation must encompass provisions including the right to information, the use of mechanisms of the IoT, prohibition or restriction regarding the use of mechanisms of the IoT, rules on IT security legislation, and the establishment of a task force doing research on the legal challenges of the IoT (Weber, 2010).

In all instances, legislative and regulatory measures should concomitantly raise the level of risk perceived by a criminal, and decrease the favourable context to perpetrate an illegal action (Grobler et al., 2012). Equally, legislative and regulatory measures should also raise the risk perceived by users who assume a role of ignorance in terms of applying specified technical measures. To this extent, research by Raschke et al. (2017b) discusses the designing the General Data Protection Regulation (GDPR)-compliant and usable privacy dashboard to address the uncertainty around how to deal with the existing technologies to conform to the 2016 GDPR law. They designed a dashboard to enable and ease the execution of data privacy rights as per GDPR. The dashboard provides the interactions between the data subjects and data controller. The dashboard is designed using Nielson’s usability engineering lifecycle.

Despite the presence of policy frameworks, the behaviors of users and organizations make the cyber security system fail. Many organizational policies support a fairly static approach, where the same policy is used for several years without considering the evolution of the cyber threat landscape. Furthermore, organizations to a large extent follow a tick the box approach, with no real consideration for the real intention in terms of cyber security. Their inability to learn and adapt dynamically opens the door for advanced threats. These weaknesses in security governance create systemic control gaps and vulnerabilities (Julisch, 2013). The recommendation is to move away from an existing over reliance on IT and rather focus on the interaction between system and system users.

The next section investigates the usability component in more detail.

## 5 Usability

**FIGURE 4:**
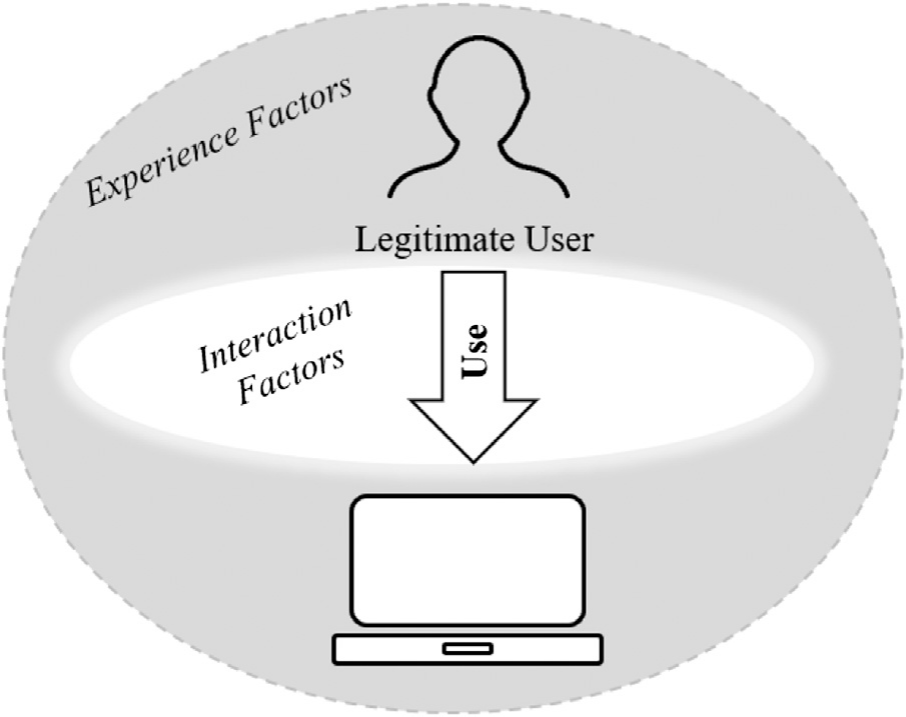
Cyber security: Usability perspective.

Despite significant investments, there are still major weaknesses in cyber security, especially in terms of true adoption of usable security. Although humans are the most active user in terms of cyber security measures, as opposed to automated system security measures that also require some level of human interaction, Julisch (2013) argues that most security investments are focused on developing technical solutions, and these technical solutions alone cannot solve the problem. We posit that decisions concerning security is based on user intuition rather than data and argues that none of the security solutions where humans are involved (e.g., Android permission interfaces) are designed based on user studies. Unless this problem is addressed and tools are developed based on user studies, it is difficult to produce usable security tools. In most of the components of usability discussed, the lack of consideration for the human element and human behavior poses some barrier to successful implementation. Figure 4 demonstrates the usability view where the system adapts to the user’s expectations and requirements in terms of cyber security. In this section, we review the two usability components of cyber security: experience factors and interaction factors.

### 5.1 Experience Factors

Experience is inherent to usability, with usability directly contributing to the overall experience of a user. The success of behavioral analytics within cyber security is therefore to make cyber security personal to the user, i.e., the system is designed to not only defend itself and the legitimate user against malicious attacks, but also adapting to serve the user in its using of the system. Stobert and Biddle (2014) recommend that successful behavioral analytics approaches should involve perceptive behavior and careful self-management of user resources. To this end, they developed a password lifecycle that evolves with the user and changing security requirements to harness existing user behavior while limiting negative consequences. Users must feel a sense of personal loss if their account is compromised to fully understand the negative consequences of their own poor cyber security behavior (Tam et al., 2010).

In terms of functionality, information dissemination is not fully adapted toward humans. It is observed that security related information are not always readily available for users (Iacono et al., 2017) and that security issues are not communicated in general public terms. Problems of appropriate response to cyber events are further exacerbated when security technology is perceived as an obstacle to the user. The user may be overwhelmed by difficulties in security implementation, or may mistrust, misinterpret or override security configurations (Pfleeger and Caputo, 2011) in response to a negative user experience. This ‘resistance behavior’ is often visible when users are faced with a mandatory password change, or when additional steps need to be taken to ensure adequate security, such as scrutinising mobile app permissions before installation or carrying around additional hardware required for multi-factor authentication (Krol et al., 2015). Braz and Robert (2006) did an exhaustive study on the usability of multi-factor authentication and found it significantly strengthens security through redundancy, but that it has a noticeable negative impact on usability. Krol et al. (2015) specifically note that when security is not the primary task at hand, users are often frustrated by complex authentication tasks.

People are increasingly using their smart phones to engage with the Internet. This has lead to an increasing interest in the personalization of the security of smart spaces. To this end, Greaves and Coetzee (2017) propose a proximity based local personal smart space. The idea is to provide context driven access control to shared content on mobile devices. Similarly, Lebeck et al. (2018) studied the security, privacy and safety concerns on immersive augmented reality (AR) technology by performing user studies. Unlike other studies where the focus has been on individual users with a device, this study focuses on multiple users, each with their own AR device, but sharing the same space and interacting with the same embedded objects. The study highlighted the need of human centric design for the security, privacy and safety in AR applications.

For a positive user experience and an ideal authentication process, Krol et al. (2015) suggest fewer steps and no requirement for additional authentication tokens. To enhance this experience, Aumi and Kratz (2014) developed a biometric authentication technique, AirAuth, that uses in-air gesture input to authenticate users. This technique requires only a low amount of computational power and is deployable on embedded or mobile hardware. To some extent, traditional password and PIN-based authentication is a compromise between the level of security and experience. In contrast with many of the traditional authentication methods, the gesture based authentication system’s security is positively aligned with experience and excitement.

### 5.2 Interaction Factors

Human centric cyber security becomes more important with the emergence of cyber physical systems as it changes the way in which users interact with the physical world. As cyber space becomes an intermediary between humans and the physical world, security solutions need to be more understandable and usable (Denning et al., 2009; Rajkumar et al., 2010), and the interaction needs to be intuitive and user-focused.

Dourish et al. (2004) argue that security solutions should consider specific interactions factors that could improve human’s using of computers (Figure 4). Both Haack et al. (2009) and Tyworth et al. (2013) make a case for mixed-initiative cyber security where the focus is put on humans-in-the-loop, i.e., the human (Section 3) and the cyber system (Section 4) working together toward usability. This humans-in-the-loop concept is more commonly referred to as orchestration, where the aim is to make all levels of user feel comfortable to interact with the system. In this context, human centric cyber security requires fully integrated interaction between the system and the user, where the user’s behavior is reflected in how the system interacts with them, i.e., the system generates a different interface with varied content, dependent on the input given by the user.

The evolvement and more ubiquitous use of IoT has had a ripple effect on how and where users integrate with technology. Not only is IoT a fruitful application of technology that can, to a large extent, make the life of the user easier, but conversely, the merging threat of IoT presented in security, privacy and safety environments as the physical objects are now interacting with cyber space. Riahi et al. (2014) therefore developed a framework for IoT security where the human takes a central role. The framework uses four key components: people, intelligent objects (sensors and actuators), technological ecosystem (communication, protocols, systems), and process (interactions between them). These components are discussed next, with the exception of the people component, as this was already discussed in Section 3.

#### 5.2.1 Intelligent Objects

Although smart phones add value and context to human centric interaction, these interfaces are not obvious to users and can be used to extract private information in a stealthy way. The challenge is to design a usable permission interface from a privacy point of view. Gerber et al. (2017) specifically studied two key factors in designing usable privacy, understandability and comprehensiveness in the context of Android apps. The understandability increases the quality of information provided to users, whereas comprehensiveness increases the quality of decision made by users. The right balance between these two factors is a challenge. The authors proposed a permission-granting interface, called COPING (COmprehensive PermIssioN Granting), and compare with other interfaces. The aim is to effectively inform users to make quality decisions.

#### 5.2.2 Technological Ecosystem

The ecosystem looks at the integration of all components to a cohesive unit. Although there are many benefits to such an integrated ecosystem, this connectedness increases the cyber attack vector. For example, Bonneau and Preibusch (2010) observed that more secure sites are vulnerable via less secure sites since many users either use the same password on many websites or the systems incorporate prioritised third-party access in their system design. Particularly in the area of cyber interaction, we consider emerging technology such as social media, cloud computing, pervasive mobile computing, big data and IoT. Research by Wijayarathna et al. (2017) specifically considered the interaction of system developers and the Application Programmming Interfaces (APIs) that they use. They found that usability issues that exist in security APIs cause programmers to embed those security APIs incorrectly in the applications that they develop. This results in introduction of security vulnerabilities to those applications. To address this problem, they have implemented a usability evaluation methodology by using cognitive dimensions to evaluate the usability of security APIs.

#### 5.2.3 Process

Linked to the need for a positive experience, the process for security needs to be suitable for the user. An example of such a positive process is presented by Wu et al. (2018) in the development of PERSCRIPTION, a system designed to generate personalised security-centric descriptions that automatically learn users’ security concerns and linguistic preferences to produce user-oriented descriptions. Not only does this process communicate the needed system usage and security policy information (Section 4.3), but also it adapts to the user (Section 3) to provide a positive experience (Section 5.1). From the system designer perspective, Senarath et al. (2019) proposed a model that could be used by system developers to measure the privacy risk perceived by users when they disclose data into software systems. This model is derived based on the perceived privacy risk of users, based on their existing knowledge of the system and their own data.

## 6 Discussion

Based on the literature survey conducted, we concur with Adams and Sasse (1999) that it is not the user that is the weakest link. In fact, the disconnect of humans (including security experts, security system designers and implementers, as well as general end-users) forms the weakest link that should be addressed in human centric cyber security. We argue that human vulnerabilities are no longer the most prominent problem, but rather the disconnect between humans and the systems that they are depending on. It is the failure of all humans involved in the system, and not only the end-user, that should be acknowledged and integrated in a human centric solution to cyber security.

### 6.1 Application of the 3U Model

There is a definite need for technologies that can help overcome the barriers between user, usage and usability to better meet security and privacy needs. The conceptual study that we have conducted has lead to developing this new 3U model to explain the cyber security approach to be taken to ensure a full human centric focus. By defining the 3U model for human centric cyber security, we propose a paradigm shift from users as the weakest link by incorporating user centred cyber security to involve the human in the solution of system design. This model aims to put in place a baseline for human centric cyber security, ensuring that all three components are addressed.

**FIGURE 5:**
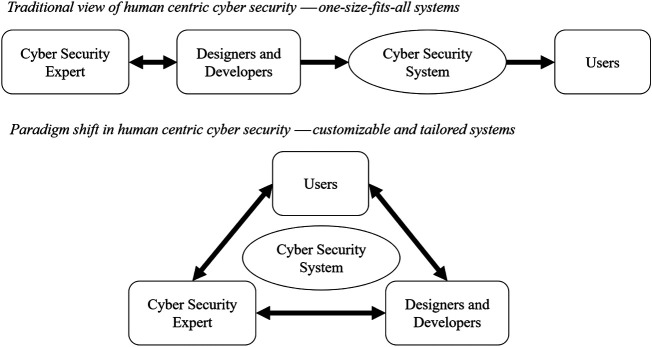
Human centric cyber security: Paradigm shift.

The 3U model is aimed at better understanding the perceptions around cyber security application, from a human centric perspective. As a mechanism designed to provide insights about the better integration of all components relevant to cyber security systems, the understanding brought about by focusing on the three components stretches beyond the traditional understanding of cyber security. This model integrates the paradigm shift for human centric cyber security to determine the relative importance that system users perceive in enabling and supporting continueds use of their systems. We argue that the approach to cyber security needs to shift from developing one-size-fits-all systems for users (top) to developing customizable and tailored systems with users (bottom), as illustrated in Figure 5. The study of cyber security should go beyond vulnerabilities of usage and usability, and encompass user vulnerabilities as well. Ultimately, technology needs to be usable by all users. To enable this, system designs should capture different user personalities within the system functionality.

The real-time nature of cyber threats requires humans not to become bottlenecks (Haack et al., 2009) and therefore, security departments need to communicate more with users in order to fully adopt a user centered design approach (Adams and Sasse, 1999). Essentially, cyber security awareness should not be targeted at general end-users (in the traditional sense, referring to the system end-user or general end-user) alone. Rather, it should be regarded as a multi-way communication among general end-users, security experts and system developers. End users need to be aware of cyber security consequences in the systems they use, i.e., *what will happen to me and my personal data if I do not use a strong and secure password and how may this affect the functionality of the system?* Equally, the cyber system (through the system designer) needs to be aware of user factors to accommodate user needs in delivering usable cyber security systems, i.e., *have I considered all types of users who may interact with this system?* Furthermore, experts also need to be aware of user traits as that would help identify user related vulnerabilities. Such multi-way communication is the only way to achieve not only system centric or user centric, but also true human centric cyber security.

### 6.2 Case Study

The application of the 3U model is an iterative process where the design components combine to form a holistic modelling approach to designing and developing a system. Each of these components, with its own set of inclusions, exclusions and focus areas, would be separately built to ensure the relevant cyber security aspects are sufficiently considered to present an integrated model. The context would vary according to the specific system being implemented. We present a case study to demonstrate how the application of the 3U model could provide a human centric perspective in cyber security. We consider a generic instance of a national government service system, and map the example of authentication requirement against the respective 3U component elements. In this example, the 3U model provides a conceptual viewpoint and focuses on better understanding all human centric cyber security components, and not on specific security areas and risks, or how the system needs to adapt. A number of questions are suggested for each of the three components, to explore critical indicators that could be incorporated in each component. The listed questions are not exhaustive, but rather a starting point for an iterative design process. This process is intended to guide the co-design and development of a more human centric focused system.

#### 6.2.1 User

In this example, we consider *general end-users*, *IT managers* and *system developers* among various legitimate users who would interact with the system. When considering this component for users consider that: *if a user does not feel that a system is suited in terms of what it would like to see in the system, the likelihood of the system being fully explored and used as intended will decrease*. For example, if a government service system requires authentication and only multi factor authentication using smartphones is provided, then a newly migrated refugee who does not have access to a smartphone or a person with disability who is unable to use a smartphone, is less likely to use the service through the digital platform.

- Demography and culture-A baseline for the user profile can help identify factors that contribute to increased online risk behaviors and thereby develop mitigation strategies.
  -Who are the end-users, what are their demography, cultural background?
  -Do age, gender, education, etc. have any impact on the way the user might use the system?
  -Do IT managers have an appropriate and secure way to enable access to end-users who may have exceptional needs?
  -Do system designers have matching user profiles or access to people having such user profiles?
- Situational awareness-Education and training may improve users’ awareness and perception pertaining to the cyber security situation.
  -Does the user have a basic understanding of Internet security?
  -Can users relate to cyber security issues within their physical world?
  -Can educators adapt training materials to trainees’ past experiences?
- Psychology and behavior-Systems addressing psychological and security needs perform better compared to systems that address only security needs.
  -Would a user be switching between different roles, i.e., moving from legitimate user to malicious user, or from end-user to system designer?
  -Is the system open to a user taking recreational risks?
  -Is there a mismatch between the perceptions of users and the reality of system?
- Cognitive factors-Understanding users’ cognitive factors may help select and/or develop systems that work for them.
  -Is the security model easy to relate to everyday experiences of users?
  -Are any user mental models applicable with regard to the security perception of the system?

#### 6.2.2 Usage

This component is concerned with functional and security aspects of the system, specifically with technological and non-technological measures. It determines a baseline of the user’s expectations, intention and likelihood to continuously use the system for the intended purpose: *if a user feels that the purpose of a system is not suited to its needs, or the security measures put in place are overly restrictive, the likelihood of the system being fully utilised will decrease*. For example, if the system has multiple usage such as providing public information, discussion forums and personalised information for users, having a single authentication mechanism might not be appropriate.

- Functional measures-A baseline of key functional areas provided by the system should be identified to prioritize value add functional visibility as well as to provide an appropriate level of security.
  -What are the key functions of this system and do all functions require the same level of security?
  -Is there an alternate, less restrictive way of providing the same security to achieve functional usage?
  -Should different authentication mechanisms be provided to different types of users?
- Technical measures-Transparent security mechanisms should be implemented to provide built in security.
  -Does a user need to sign-in again if it is already in a secure network zone?
  -Would authentication through an enterprise single sign-on or third party account, such as a Google or Facebook account, be suitable?
  -Are the use of password managers encouraged in this system?
- Legislation, regulations and policies-The inclusion of legislative elements that would support appropriate minimum levels of cyber security within systems.
  -Are there any specific laws or regulations that dictate specific requirements in terms of usage?
  -Are there organisation policies that ensure proper system usage or may influence the choice of system design?

#### 6.2.3 Usability

The usability component focuses on how well the system can be used by the actual user. From the system design perspective, this component is critical in providing added value and design features with the premise that: *if the user finds benefit in using the system and associates positive experiences with its use, the likelihood of the system being used for a longer term will increase*. For example, if the system focuses on a minimalistic design approach and provide console based access, it may offer limited appeal to users who require extended graphical user interfaces to connect with the system.

- Experience factors-Factors that contribute to a positive experience would assist in developing a human centric focus.
  -Do users experience a significant loss of anonymity in the modern technology landscape?
  -Do users realize that they are leaving digital traces while consuming modern media and that such traces are archival?
  -What are the users’ stance on anonymity vs. usability?
- Interaction factors-Factors that support the interaction between the user and the system are better placed to provide usable experiences.
  -Does increased usage of smartphones to access personal and corporate data introduce additional usability issues as a result of device-level authentication schemes, particularly in terms of the relative small sized keyboards on smartphones?
  -Can system components be better integrated to provide better experience to human users?

## 7 Conclusion and Future Directions

Human centric system design is gaining momentum. Since the original introduction of human computer interaction in 1960 (Renaud and Flowerday, 2017), significant progress has been made in terms of the evolving concept of usability to integrate a concrete commitment to value human activity and experiences as the primary driver in technology (Carrol, 2013). The shift from a pure focus on usage toward a consideration for human behavior is a strong move toward human centric cyber security, and will contribute to better usage and usability of the systems.

The work presented here provides a baseline for the understanding and incorporation of a holistic human centric cyber security system design, and not an exhaustive overview of technology advances and automation within the field of cyber security. By focusing on three unique components, this review aims to incorporate the concept of invisible security, that it, the automation of selective cyber security tasks, whilst maintaining the ability of the human user to remain in the loop and utilise collaborative intelligence between the human and the technology to further the cyber security domain. Historically, users are considered as the weakest link within cyber security (Tsinganos et al., 2018), although they are not always to blame for security compromises (Adams and Sasse, 1999). The effectiveness of too many cyber security measures are questioned in the wake of hard hitting security events, and there is a definite stigma attached to users in cyber space, particularly as a result of numerous cyber security events and breaches following on poor cyber behaviors of users. By conducting this review on human centric cyber security, our aim was to better understand the ecosystem of this domain. By investigating current literature focusing on the 3U components, we identified areas of research that already strongly linked with a human centric focus, such as usable authentication, but also identified areas that are not yet generally associated with a humanistic focus, in particular data sharing with a privacy and security focus.

Many studies emphasize the user components that consider the individual’s demographic and skills differences, personality and cognitive burdens or biases for negative feedings toward security. When looking into the usage domain, we realized that the users of a cyber security system are not only the lay users, but also experts who deploy the system and developers who try to defend against adversaries. We conclude to consider all these humans involved in the cyber security ecosystem as a part of human centric cyber security studies. In addition, we propose the 3U framework can help better understand the interconnection between human and cyber security, develop usable systems and lift the users to rather be the strongest link within the cyber security space. Accordingly, HCI could be streamlined to ensure a positive experience that integrates both the system user and functional system usage. We recommend that future human centric cyber security research and design focus on facilitating collaboration among all humans who form the ecosystem of cyber security. This may help avoid a large portion of cyber security events, making a safer and more security online environment.

Through the collaborative design and separate development, our proposed 3U model should be considered as the initial step toward developing effective cyber security technologies and their better adoption through the sustained adherence and continuous engagement of the extended human centric cyber security domain. The detailed review of building these components is a future extension of this initial exploratory literature survey. Further empirical research work is needed to ensure the validity of the detailed components, and to test whether the application of the 3U components would address the issues of cyber security technology adoption and the posed security dilemma. Future work will also include detailed discussion on how the diverse components of the 3U model can be specifically utilized to develop a fully integrative cyber security system that would encourage adaptive behaviors across all components to a better sustained and collaboratively secure environment. Ultimately, a fully human centric cyber security system would reduce the vulnerability of systems, reduce the need for reactive cyber security training, limit the scope of the security dilemma, and encourage invisible security, whilst keeping humans in the loop.

## Data Availability

The original contributions presented in the study are included in the article/Supplementary Material, further inquiries can be directed to the corresponding author.

## References

[B1] Abbott J. Garcia V. (2015). Password differences based on language and testing of memory recall. NNGT Int. J. Inf. Security 2, 1–6. 10.13140/RG.2.2.30770.71362

[B2] Acar Y. Backes M. Fahl S. Garfinkel S. L. Kim D. Mazurek M. L. (2017). Comparing the usability of cryptographic APIs. IEEE Symp. Sec. Priv. 2017, 154–171. 10.1109/SP.2017.52

[B3] Adams A. Sasse M. (1999). Users are not the enemy. Commun. ACM 42, 40–46. 10.1145/322796.322806

[B4] Adams M. Makramalla M. (2015). Cybersecurity skills training: an attacker-centric gamified approach. Tech. Innovation Manag. Rev. 5, 5–14. 10.22215/timreview861

[B5] Agarwal L. Khan H. Hengartner U. (2016). “Ask me again but don’t annoy me: evaluating re-authentication strategies for smartphones,” in Twelfth symposium on usable privacy and security (SOUPS 2016), Denver, CO, June 23, 2016 (Berkeley, CA: USENIX Association), 221–236.

[B6] Aumi M. T. I. Kratz S. G. (2014). “AirAuth: evaluating in-air hand gestures for authentication,” in Mobile HCI. Editor Salam. A. (New York, NY: ACM SIGCHI) 309–318. 10.1145/2559206.2574797

[B7] Bonders M. Slihte J. (2018). “A practical approach to teaching information technology infrastructure management,” in 2018 59th international scientific conference on information technology and management science of Riga Technical University (ITMS), Riga, Latvia, October 10–12, 2018, 1–5. 10.1109/ITMS.2018.8552960

[B8] Bonneau J. Preibusch S. (2010). “The password thicket: technical and market failures in human authentication on the web,” in The ninth workshop on the economics of information security, Cambridge, MA, June 7-8, 2010, 1–49.

[B9] Bosnjak L. Brumen B. (2016). “What do students do with their assigned default passwords?,” in 2016 39th international convention on information and communication technology, electronics and microelectronics (MIPRO), May 30–June 3, 2016, Opatija, Croatia, 1430–1435. 10.1109/MIPRO.2016.7522364

[B10] Braz C. Robert J.-M. (2006). “Security and usability: the case of the user authentication methods,” in Proceedings of the 18th conference on l’interaction homme-machine, Paris, France, April 18, 2006 (ACM), 199–203.

[B11] Camp L. J. Abbott J. Chen S. (2016). “Cpasswords: leveraging episodic memory and human-centered design for better authentication,” in 2016 49th Hawaii international conference on system sciences (HICSS), Koloa, HI, January 5–8, 2016 (IEEE), 3656–3665.

[B12] Camp L. J. Grobler M. Jang-Jaccard J. Probst C. Renaud K. Watters P. (2019). “Conceptualizing human resilience in the face of theglobal epidemiology of cyber attacks,” in Proceedings of the 52nd Hawaii international conference on system sciences (HICSS-52), Grand Wailea, HI, January 8–11, 2019 [abstract].

[B13] Carrol J. M. (2013). The Encyclopedia of human-computer interaction (the interaction design foundation). 2nd Edn. Denmark: IDF, Chap. 2.

[B14] Cavelty M. D. (2014). Breaking the cyber-security dilemma: aligning security needs and removing vulnerabilities. Sci. Eng. Ethics 20, 701–715. 10.1007/s11948-014-9551-y 24781874

[B15] Chiasson S. van Oorschot P. C. Biddle R. (2006). “A usability study and critique of two password managers,” in USENIX security symposium, Vancouver, BC, July 31–August 4, 2006, 221–236.

[B16] Conti M. Passarella A. (2018). The internet of people: a human and data-centric paradigm for the next generation internet. Comput. Commun. 131, 51–65. 10.1016/j.comcom.2018.07.034

[B17] Da Veiga A. (2016). “A cyber security culture research philosophy and approach to develop a valid and reliable measuring instrument,” in SAI Computing Conference (IEE), London, United Kingdom, July 15, 2016 [abstract].

[B18] De Donno M. Dragoni N. Giaretta A. Spognardi A. (2018). DDoS-capable IoT malwares: comparative analysis and Mirai investigation. Security Commun. Networks 2018 (4), 1–30. 10.1155/2018/7178164

[B19] Denning T. Matuszek C. Koscher K. Smith J. R. Kohno T. (2009). A spotlight on security and privacy risks with future household robots: attacks and lessons. UbiComp 2009, 105–114. 10.1145/1620545.1620564

[B20] Devillers M. (2010). “Analyzing password strength,” in Radboud university Nijmegen Tech. Rep.. Nijmegen, Netherlands: Radboud University, Vol. 2.

[B21] Dimensional Research (2018). The risk of social engineering on information security: a survey of it professionals. Friuli-Venezia Giulia, Italy: Stamx.NET.

[B22] Dourish P. Grinter R. E. de la Flor J. D. Joseph M. (2004). Security in the wild: user strategies for managing security as an everyday, practical problem. Pers Ubiquit Comput. 8, 391–401. 10.1007/s00779-004-0308-5

[B23] Dunphy P. Vines J. Coles-Kemp L. Clarke R. Vlachokyriakos V. Wright P. (2014). “Understanding the experience-centredness of privacy and security technologies,” in Proceedings of the 2014 workshop on new security paradigms, Victoria, BC, September 15–8, 2014, 83–94. [abstract].

[B24] Felt A. P. Ha E. Egelman S. Haney A. Chin E. Wagner D. (2012). “Android permissions: user attention, comprehension, and behavior,” in Proceedings of the eighth symposium on usable privacy and security, Washington, DC, July 11–13, 2012 (ACM), 3.

[B25] Florencio D. Herley C. (2007). “A large-scale study of web password habits,” in Proceedings of the 16th international conference on World Wide Web, Banff, AL, May 08–12, 2007 (ACM), 657–666.

[B26] Gao X. Yang Y. Liu C. Mitropoulos C. Lindqvist J. (2018). “Forgetting of passwords: ecological theory and data,” in 27th USENIX Security Symposium, Baltimore, MD, August 15–17, 2018. 10.1145/3213846.3229502

[B27] Gcaza N. von Solms R. Grobler M. van Vuuren J. J. (2017). A general morphological analysis: delineating a cyber-security culture. Ics 25, 259–278. 10.1108/ICS-12-2015-0046

[B28] Gerber P. Volkamer M. Renaud K. (2017). The simpler, the better? Presenting the COPING Android permission-granting interface for better privacy-related decisions. J. Inf. Security Appl. 34, 8–26. 10.1016/j.jisa.2016.10.003

[B29] Giacobe N. A. Xu S. (2011). Geovisual analytics for cyber security: adopting the geoviz toolkit. IEEE VAST 2011, 315–316. 10.1109/VAST.2011.6102491

[B30] Greaves B. Coetzee M. (2017). Access control for secure information sharing in smart content spaces. J. Inf. Security Appl. 34, 63–75. 10.1016/j.jisa.2016.12.002

[B31] Grobler M. Flowerday S. Solms R. V. Venter H. (2011). “Cyber awareness initiatives in South Africa: a national perspective,” in First IFIP TC9/TC11 Southern African cyber security awareness workshop, Gaborone, Botswana, May 12, 2011 (SACSAW11), 32–41.

[B32] Grobler M. van Vuuren J. J. Leenen L. (2012). “Implementation of a cyber security policy in South Africa: reflection on progress and the way forward,” in ICT critical infrastructures and society—10th IFIP TC 9 international conference on human choice and computers, HCC10 2012. Heidelberg, Germany: Springer, 215–225. 10.1007/978-3-642-33332-3_20

[B33] Gunson N. Marshall D. Morton H. Jack M. (2011). User perceptions of security and usability of singlefactor and two-factor authentication in automated telephone banking. Comput. Security 30, 208–220. 10.1016/j.cose.2010.12.001

[B34] Haack J. N. Fink G. A. Maiden W. M. Mckinnon D. Fulp E. W. (2009). “Mixed-initiative cyber security: putting humans in the right loop,” in The eighth international joint conference on autonomous agents and multiagent systems and the first international workshop on mixed-initiative multiagent systems (MIMS), Istanbul, Turkey, May 4–8, 2015 (ACM).

[B35] Hadlington L. Murphy K. (2018). Is media multitasking good for cybersecurity? exploring the relationship between media multitasking and everyday cognitive failures on self-reported risky cybersecurity behaviors. Cyberpsychology, Behav. Soc. Networking 21, 168–172. 10.1089/cyber.2017.0524 PMC5882175 29638157

[B36] Hassenzahl M. Diefenbach S. Göritz A. (2010). Needs, affect, and interactive products-facets of user experience. Interacting Comput. 22, 353–362. 10.1016/j.intcom.2010.04.002 Modelling user experience-an agendafor research and practice

[B37] Higashino T. Uchiyama A. (2012). A study for human centric cyber physical system based sensing-toward safe and secure urban life. ISIP 2012, 61–70. 10.1007/978-3-642-40140-4_7

[B38] Holland N. (2020). The human-centric cybersecurity stance. Available at: https://www.bankinfosecurity.com/human-centric-cybersecurity-stance-a-13897 (Accessed July 08, 2020). 10.1287/2961bfc6-3c5b-481a-ae7c-47edf9c88831

[B39] Howard N. Cambria E. (2013). Intention awareness: improving upon situation awareness in human-centric environments. Hum. Cent. Comput. Inf. Sci. 3, 1–17. 10.1186/2192-1962-3-9

[B40] Iacono L. L. Gorski P. L. Grosse J. Gruschka N. (2017). Signalling over-privileged mobile applications using passive security indicators. J. Inf. Security Appl. 34, 27–33. 10.1016/j.jisa.2016.11.006

[B41] Jeong J. Grobler M. Chamikara M. Rudolph C. (2019). “Fuzzy logic application to link national culture and cybersecurity maturity,” in IEEE 5th International Conference on Collaboration and Internet Computing (CIC), Los Angeles, CA, December 12–14, 2019, 330–337. 10.1109/CIC48465.2019.00046

[B42] Jones D. Endsley M. (1996). Sources of situation awareness errors in aviation. Aviat Space Environ. Med. 67, 507–512. 8827130

[B43] Julisch K. (2013). Understanding and overcoming cyber security anti-patterns. Computer Networks 57, 2206–2211. 10.1016/j.comnet.2012.11.023

[B44] Kassicieh S. Lipinski V. Seazzu A. F. (2017). “Designing a GDPR-compliant and usable privacy dashboard,” in IFIP international summer school on privacy and identity management privacy and identity 2017: privacy and identity management, Ispra, Italy, September 4–8, 2017, 221–236.

[B45] Kassicieh S. Lipinski V. Seazzu A. F. (2015). “Human centric cyber security: what are the new trends in data protection?,” in Management of Engineering and Technology (PICMET), 2015 Portland International Conference on (IEEE), 1321–1338.

[B46] Kluge E.-H. W. (2007). Secure e-health: managing risks to patient health data. Int. J. Med. Inform. 76, 402–406. 10.1016/j.ijmedinf.2006.09.003 17084665

[B47] Komanduri S. Shay R. Kelley P. G. Mazurek M. L. Bauer L. Christin N. (2011). “Of passwords and people: measuring the effect of password-composition policies,” in Proceedings of the SIGCHI conference on human factors in computing systems, Austin, TX, May 5–10, 2012 (ACM), 2595–2604.

[B48] Kraus L. Wechsung I. Möller S. (2017). Psychological needs as motivators for security and privacy actions on smartphones. J. Inf. Security Appl. 34, 34–45. 10.1016/j.jisa.2016.10.002

[B49] Krol K. Philippou E. De Cristofaro E. Sasse M. A. (2015). “They brought in the horrible key ring thing!” analysing the usability of two-factor authentication in UK online banking,”in The 2015 network and distributed system security (NDSS) symposium: USEC workshop, San Diego, CA, February 8–11, 2015. Reston, US: The Internet Society. 123–140. 10.14722/usec.2015.23001

[B50] Labuschagne W. A. Eloff M. (2014). “The effectiveness of online gaming as part of a security awareness program,” in 13th European conference on cyber warfare and security (ECCWS), Piraeus, Greece, July 3–4, 2014, (Academic Conferences Limited), 125–132.

[B51] Lebeck K. Ruth K. Kohno T. Roesner F. (2018). Towards security and privacy for multi-user augmented reality: foundations with end users,” in IEEE symposium on security and privacy, San Francisco, CA, May 24, 2018, 392–408. [abstract].

[B52] Leenen L. Aschman M. Grobler M. van Heerden A. (2018). “Facing the culture gap in operationalising cyber within a military context,” in 13th international conference on cyber warfare and security (ICCWS 2018), Washington, DC, March 8–9, 2018 Academic Conferences and Publishing International Ltd., 387–394.

[B53] Liu Z. Hong Y. Pi D. (2014). A large-scale study of web password habits of Chinese network users. J. Soc. Work 9, 293–297. 10.4304/jsw.9.2.293-297

[B54] Moller A. Michahelles F. Diewald S. Roalter L. Kranz M. (2012). “Update behavior in app markets and security implications: a case study in Google Play” in Proceedings of the 3rd international workshop on research in the large, San Francisco, September 2012. 3–6.

[B55] Moon D. Pan S. B. Kim I. (2015). Host-based intrusion detection system for secure human-centric computing. J. Supercomput. 72, 2520–2536. 10.1007/s11227-015-1506-9

[B56] Newman M. E. Forrest S. Balthrop J. (2002). Email networks and the spread of computer viruses. Phys. Rev. E 66, 1–4. 10.1103/PhysRevE.66.035101 12366169

[B57] Pfleeger S. L. Caputo D. D. (2011). Leveraging behavioral science to mitigate cyber security risk. Comput. Security 31, 597–611. 10.1016/j.cose.2011.12.010

[B58] Pilar D. R. Jaeger A. Gomes C. F. A. Stein L. M. (2012). Passwords usage and human memory limitations: a survey across age and educational background. PLoS One 7, 2–7. 10.1371/journal.pone.0051067 PMC3515440 23227232

[B59] Qu Z. Rastogi V. Zhang X. Chen Y. Zhu T. Chen Z. (2014). “Autocog: measuring the description-to-permission fidelity in android applications,”in Proceedings of the 2014 ACM SIGSAC conference on computer and communications security, Scottsdale, AZ, November 3–7, 2014 ACM, 1354–1365.

[B60] Rajkumar R. Lee I. Sha L. Stankovic J. A. (2010). “Cyber-physical systems: the next computing revolution,” in DAC, Anaheim, CA, June 13-18, 2010, 731–736.

[B61] Raschke P. Küpper A. Drozd O. Kirrane S. (2017a). “Designing a gdpr-compliant and usable privacy dashboard,”in IFIP international summer school on privacy and identity management. Berlin, Germany: Springer, 221–236.

[B62] Raschke P. Kupper A. Drozd O. Kirrane S.. (2017b). “Designing a gdpr-compliant and usable privacy dashboard,” in IEEE symposium on security and privacy, San Jose, CA, May 22–26, 2017, 392–408.

[B63] Renaud K. Flowerday S. (2017). Contemplating human-centred security and privacy research: suggesting future directions. J. Inf. Security Appl. 34, 8–26. 10.1016/j.jisa.2017.05.006

[B64] Riahi A. Natalizio E. Challal Y. Mitton N. Iera A. (2014). “A systemic and cognitive approach for IoT security,” in International conference on computing, networking and communications (IEEE), Onolulu, Hawaii, February 3–6, 2014. 10.1109/iccnc.2014.6785328

[B65] Ruoti S. Monson T. Wu J. Zappala D. Seamons K. (2017). “Weighing context and trade-offs: how suburban adults selected their online security posture,” in Proceedings of the thirteenth symposium on usable privacy and security (SOUPS 2017), Santa Clara, CA, July 12–14, 2017 (Berkeley, CA: USENIX Association). 10.1109/secdev.2017.20

[B66] Ruoti S. Roberts B. Seamons K. (2015). “Authentication melee: a usability analysis of seven web authentication systems,” in Proceedings of the 24th international conference on World Wide Web, Florence, Italy, May 18–22, 2015 (International World Wide Web Conferences Steering Committee), 916–926.

[B67] Senarath A. Grobler M. Arachchilage N. A. G. (2019). A model for system developers to measure the privacy risk of data,” in Proceedings of the 52nd Hawaii international conference on system sciences (HICSS-52), Maui, HA, January 8–11, 2019. 10.24251/hicss.2019.738

[B68] Simko L. Lerner A. Ibtasam S. Roesner F. Kohno T. (2018). Computer security and privacy for refugees in the United States. IEEE Symp. Security Privacy 2018, 373–387. 10.1109/SP.2018.00023

[B69] Stobert E. Biddle R. (2014). “The password life cycle: user behaviour in managing passwords,” in Tenth symposium on usable privacy and security, Menlo Park, CA, July 9–11, 2014 (Berkeley, CA: USENIX Association), 243–255.

[B70] Swart I. Irwin B. Grobler M. (2014). “On the viability of pro-active automated PII breach detection: a South African case study,” in Proceedings of the Southern African institute for computer scientist and information technologists annual conference 2014 (SAICSIT), Centurion, South Africa. (New York, NY: ACM). 10.1145/2664591.2664600

[B71] Tam L. Glassman M. Vandenwauver M. (2010). The psychology of password management: a tradeoff between security and convenience. Behav. Inf. Tech. 29, 233–244. 10.1080/01449290903121386

[B72] Tan M. Aguilar K. (2012). Risk perceptions of cyber-security and precautionary behaviour. Info Mngmnt Comp. Security 20, 364–381. 10.1108/09685221211286539

[B73] Thomas J. Galligher G. (2018). Improving backup system evaluations in information security risk assessments to combat ransomware. Comput. Inf. Sci. 11. 10.5539/cis.v11n1p14

[B74] Tischer M. Durumeric Z. Foster S. Duan S. Mori A. Bursztein E. (2016). Users really do plug in USB drives they find. IEEE Symp. Security Privacy 2016, 306–319. 10.1109/SP.2016.26

[B75] Tsinganos N. Sakellariou G. Fouliras P. Mavridis I. (2018). Towards an automated recognition system for chat-based social engineering attacks in enterprise environments,” in Proceedings of the 13th international conference on availability, reliability and security (ACM), Hamburg, Germany, August 27–30, 2018, 53.

[B76] Tyworth M. Giacobe N. A. Mancuso V. F. McNeese M. D. Hall D. L. (2013). A human-in-the-loop approach to understanding situation awareness in cyber defence analysis. EAI Endorsed Trans. Security Saf. 1, e6. 10.4108/trans.sesa.01-06.2013.e6

[B77] Ur B. Noma F. Bees J. Segreti S. M. Shay R. Bauer L. (2015). “I added ‘!’ at the end to make it secure”: observing password creation in the lab,” in Eleventh symposium on usable privacy and security (SOUPS 2015), Santa Clara, CA, July 14, 2015. (Berkeley, CA: USENIX Association), 123–140.

[B78] Van Schaik P. Jeske D. Onibokun J. Coventry L. Jansen J. Kusev P. (2017). Risk perceptions of cyber-security and precautionary behaviour. Comput. Hum. Behav. 75, 547–559. 10.1016/j.chb.2017.05.038

[B79] Weber R. H. (2010). Internet of Things-new security and privacy challenges. Comput. L. Security Rev. 26, 23–30. 10.1016/j.clsr.2009.11.008

[B80] Whitty M. Grobler M. Janicke H. (2020). Risks, mitigations and interventions of mass remote working during the COVID-19 pandemic. Joondalup, WA: Cyber Security Cooperative Research Centre.

[B81] Wijayarathna C. Arachchilagen N. A. G. Slay J. (2017). “Using cognitive dimensions questionnaire to evaluate the usability of security APIs,” in Proceedings of the 28th annual meeting of the psychology of programming interest group, Delft, The Netherlands, July 1–3, 2017.

[B82] Williams M. Nurse J. Creese S. (2017). “Privacy is the boring bit: user perceptions and behaviour in the internet-of-things,” in 15th International conference on privacy, security and trust (PST’2017), Calgary, AL, August 28–30, 2017. 10.1109/pst.2017.00029

[B83] Wu T. Tang L. Xu Z. Wen S. Paris C. Nepal S. (2018). Catering to your concerns: automatic generation of personalised security-centric descriptions for Android apps. CoRR abs/1805.07070.

